# Mitigation of Participant Loss to Follow-Up Using Facebook: All Our Families Longitudinal Pregnancy Cohort

**DOI:** 10.2196/10441

**Published:** 2019-02-15

**Authors:** Nikki Lee Stephenson, Erin Hetherington, Shawn Dodd, Alexander Mathews, Suzanne Tough

**Affiliations:** 1 Department of Community Health Sciences Cumming School of Medicine University of Calgary Calgary, AB Canada; 2 Department of Paediatrics Cumming School of Medicine University of Calgary Calgary, AB Canada; 3 O'Brien Centre for the Bachelor of Health Sciences Cumming School of Medicine University of Calgary Calgary, AB Canada

**Keywords:** social media, social networking, longitudinal studies, patient dropouts, cohort studies, follow-up studies, lost to follow-up

## Abstract

**Background:**

Facebook, a popular social media site, allows users to communicate and exchange information. Social media sites can also be used as databases to search for individuals, including cohort participants. Retaining and tracking cohort participants are essential for the validity and generalizability of data in longitudinal research. Despite numerous strategies to minimize loss to follow-up, maintaining contact with participants is time-consuming and resource-intensive. Social media may provide alternative methods of contacting participants who consented to follow-up but could not be reached, and thus are potentially “lost to follow-up.”

**Objective:**

The aim of this study was to determine if Facebook was a feasible method for identifying and contacting participants of a longitudinal pregnancy cohort who were lost to follow-up and re-engaging them without selection bias.

**Methods:**

This study used data from the All Our Families cohort. Of the 2827 mother-child dyads within the cohort, 237 participants were lost to follow-up. Participants were considered lost to follow-up if they had agreed to participate in additional research, completed at least one of the perinatal questionnaires, did not complete the 5-year postpartum questionnaire, and could not be contacted after numerous attempts via phone, email, or mail. Participants were considered to be matched to a Facebook profile if 2 or more characteristics matched information previously collected. Participants were sent both a friend request and a personal message through the study’s Facebook page and were invited to verify their enrollment in the study. The authors deemed a friend request was necessary because of the reduced functionality of nonfriend direct messaging at the time. If the participant accepted the study’s friend request, then a personalized message was sent. Participants were considered reconnected if they accepted the friend request or responded to any messages. Participants were considered re-engaged if they provided up-to-date contact information.

**Results:**

Compared with the overall cohort, participants who were lost to follow-up (n=237) were younger (*P*=.003), nonmarried (*P*=.02), had lower household income (*P*<.001), less education (*P*<.001), and self-identified as being part of an ethnic minority (*P*=.02). Of the 237 participants considered lost to follow-up, 47.7% (113/237) participants were identified using Facebook. Among the 113 identified participants, 77.0% (87/113) were contacted, 32.7% (37/113) were reconnected, and 17.7% (20/113) were re-engaged. No significant differences were found between those identified on Facebook (n=113) and those who were not able to be identified (n=124).

**Conclusions:**

Facebook identified 47.6% (113/237) of participants who were considered lost to follow-up, and the social media site may be a practical tool for reconnecting with participants. The results from this study demonstrate that social networking sites, such as Facebook, could be included in the development of retention practices and can be implemented at any point in cohort follow-up.

## Introduction

### Background

Prospective cohort study designs are methodologically valuable as they follow participants over time, which allows for the identification of risk factors that occur before outcomes and allow for the examination of trajectories of health and development [1]. However, this study design is particularly vulnerable to attrition over time as people move or lose interest in participating [1]. Birth and pregnancy cohorts may be particularly susceptible to participant attrition due to high mobility during this life stage and busy schedules of families with small children. In addition to introducing selection bias [2], loss of cohort participants reduces data quality, interpretability, and potential generalizability. Attrition also reduces statistical power and can threaten the accuracy of measures of association (such as odds ratios and risk ratios). Consequently, maximum follow-up rates are attempted [1,3].

The now widespread use of social networking sites, such as Facebook, can provide new opportunities for locating participants of research studies, who are difficult to track. Previous studies have used Facebook to recruit participants—specifically to identify and engage participants with rare conditions—those who are hard to reach or vulnerable [4-6]. Recruitment through Facebook can be more cost-effective than traditional methods, which is appealing to researchers [7-10]. Several studies have also successfully used Facebook to relocate participants after the original research concluded. These include a longitudinal follow-up of an intervention program for at-risk families [11], a longitudinal study of adults who used methamphetamine [12], and members of a graduating class [13]. These studies demonstrate the potential for identifying participants for whom follow-up was unplanned, who were high risk, or highly mobile. Yet, use of these methods to reduce attrition within a low-risk cohort has not been examined.

The All Our Families cohort (formerly the All Our Babies study) is a contemporary ongoing prospective community-based pregnancy cohort situated in Calgary, Alberta, Canada. A detailed overview of the study design, recruitment, eligibility, and data collection is described elsewhere [14-16]. Since recruitment, between 2008 and 2011, participants have completed 7 questionnaires; 3 questionnaires in the perinatal period (22 to 24 and 32 to 36 weeks gestation and 4 months postpartum) and 4 questionnaires in the early childhood period (1, 2, 3, and 5 years postpartum). The All Our Families cohort has utilized many methods to improve response rates and minimize loss to follow-up. These include collection of detailed and appropriate recruitment information, implementing standardized participant-tracking procedures, contacting alternative contacts, identifying 3 or more alternate contacts, increased frequency of participant contact, and offering monetary or other incentives for study participation [1,12,17-19]. However, even when all recommended strategies are implemented, a participant may not be retained in a mobile population [1]. Addresses, telephone numbers, and even emails are becoming less predictable ways of tracking participants in cohorts [20]. Recommendations to obtain personal identifying information to track participants on major databases (ie, driver’s license numbers for use with the department of motor vehicles) [20] are not feasible in every country or region as privacy laws may prohibit researchers from accessing this information without explicit prior participant consent. Therefore, in an effort to minimize selection bias and re-engage lost-to-follow-up participants, the All Our Families cohort required new methods of participant tracking.

### Objectives

The effectiveness of using social media to recontact participants in a longitudinal pregnancy cohort has not previously been examined. Among social networking sites, Facebook has emerged as one of the dominant platforms reporting 2.23 billion monthly active users as of June 30, 2018 [21]. Unlike traditional communication platforms, Facebook URLs are associated with longer periods of use compared with email addresses [12], which could potentially make retroactive tracking more accessible. Among active Facebook users, people aged between 18 and 24 years (30.9%), 25 and 34 years (22.6%), and 35 and 54 years (27.0%) comprise the largest member groups and continue to grow [22]. For this study, Facebook was chosen as a target social media platform to use as the median age of the All Our Families cohort and the typical age of Facebook users overlapped [23]. This study investigated the feasibility of 1 social networking site, Facebook, for both identifying and recontacting participants without demographic bias as part of a contemporary longitudinal pregnancy cohort situated in Calgary, Alberta, Canada.

## Methods

### Study Eligibility

**Figure 1:**
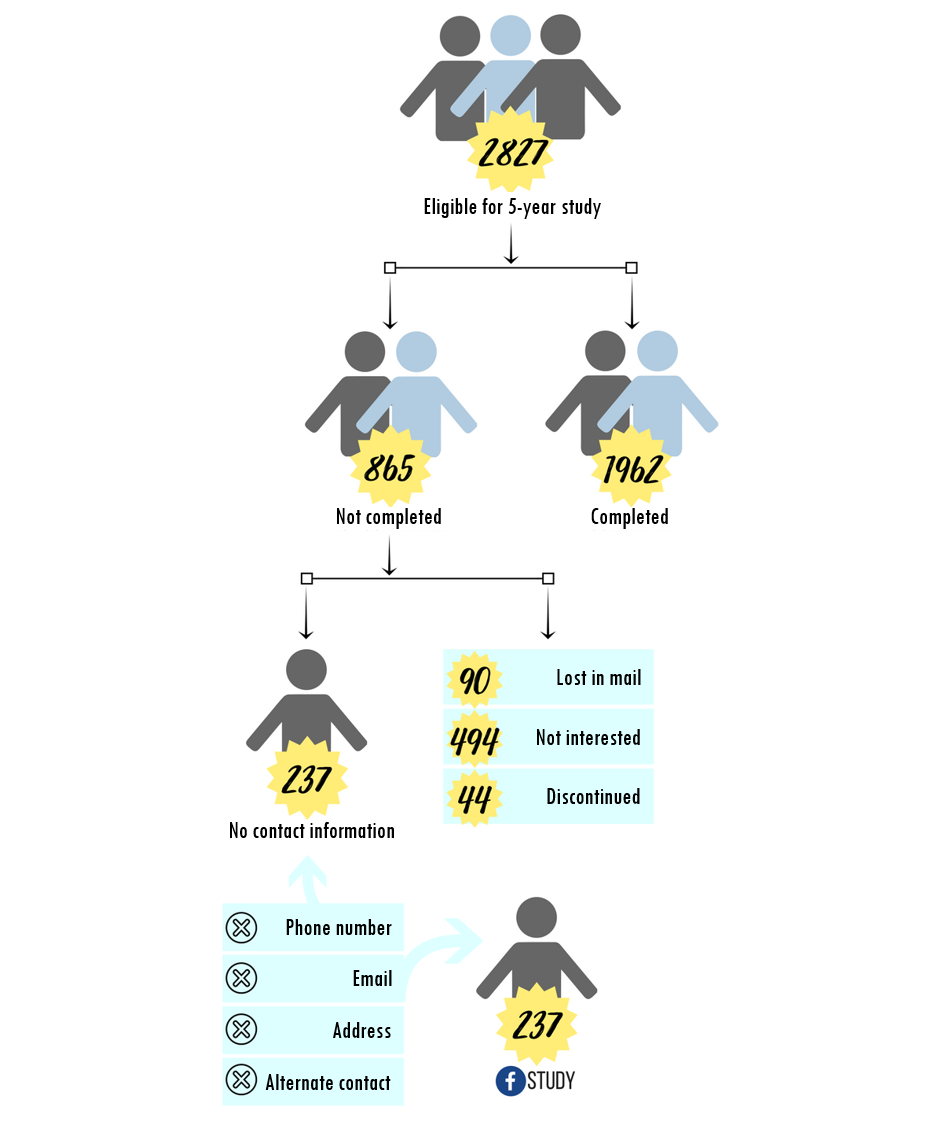
Eligibility criteria of lost to follow-up participants.

Of the 2827 mother-baby dyads who were eligible to complete the 5-year questionnaire, 237 participants were considered lost to follow-up (Figure 1). Participants were considered lost to follow-up if they had agreed to participate in additional research and completed at least one of the perinatal questionnaires, did not complete the 5-year postpartum questionnaire, and could not be contacted after numerous attempts (n=237). Participants were excluded from the re-engagement study if they had previously withdrawn or discontinued from the study or had indicated a lack of interest in the data collection wave.

### Facebook Re-Engagement Protocol

To contact participants, an All Our Families study Facebook profile was created. Throughout the course of the re-engagement study, the All Our Families Facebook profile included the study logo, a brief paragraph describing the study, the study’s contact information, and frequent updates regarding the study.

To re-engage participants, those lost to follow-up were first identified by searching the first and last name on Facebook. To verify the participants’ identity, profiles were browsed for known identifiers including birth date, home address, email address, child’s name, spouse’s name, phone number, and if the alternate contact on file was included in the participant’s friends list. If a Facebook profile contained at least two identifiers that matched data previously collected by the study, the participant was considered found on Facebook. Profiles with matching identifiers were then sent friend requests and a personalized message via Facebook’s direct messaging service, asking the profile owner to confirm their participation in the All Our Families study. The friend request was deemed necessary for direct messaging at the time of the study (May-August 2016), as messages from nonfriends were relegated to a separate section within the messages tab on Facebook's website, where messages would not prompt notifications to the user. If participants responded to the initial message, they were encouraged to contact the study team via email or telephone to update their contact information. If participants did not respond to the initial Facebook message after 2 weeks, a follow-up message was sent. A third and final follow-up message was sent to participants if they did not respond within 1 month following the initial message.

### Ethical Considerations

Social media platforms such as Facebook provide an innovative means for recruitment and retention; however, the use of social media may increase risks to participant privacy and confidentiality. This study recognized that ethical principles for ensuring privacy and confidentiality of study participant’s personal information may be affected by the use of Facebook. To protect participants’ privacy on the study’s Facebook page, the study’s Facebook privacy settings were set to “Disable posts by other people on the Page.”

Confidentiality cannot be guaranteed if a participant “shares” or “likes” the study’s page; however, by performing these actions, the participant, who has agreed to the terms and conditions of the social media site, has the ability to disclose this information as they wish according to their own privacy settings. In addition, the study’s Facebook page accepts friends who are not participants of the study, and as such, being a friend of the study does not imply that a person is a participant—only that the person may have an interest in our research on maternal child health. Nonparticipants have equal opportunity to utilize these social media functions, which further protects confidentiality as the communication is not exclusive to participants. In addition to the above safeguards, Facebook’s privacy settings were monitored on a weekly basis for potential updates. No updates or changes occurred during the data collection period. Although the study undertook precautions to protect the identity of participants, we recognized the potential risk of disclosing a participant’s identity when using social media as a public database.

The All Our Families study is a population-based cohort, with the eligibility criteria of having a child and being over the age of 18 years. The potential risk associated with the proposed re-engagement strategy was the potential harm that a person may have experienced from the disclosure, collection, and use of personal and sensitive information triggered by accepting the study’s friend request and replying to the message through the personal messaging system. This was considered to be minimal within our study population as accepting the friend request was at the discretion of the participant, who had already agreed to be contacted and had previously provided personal information to the study. If the friend request was accepted, the All Our Families study information that would have been visible was information already in the public domain about the study (eligibility criteria). The study viewed nonacceptance of the friend request or nonresponse to the direct message as the participants practicing their right to control their information while participating in social media within a public space. The study’s privacy settings were set so that only the study team could view the friend list, only mutual friends (persons who were friends with the study as well as with the newly added participant) would be visible to both the study team and to each individual friend. This was meant to maximize confidentiality for the participant as their privacy settings would determine who is able to view their association with the study. Studies that may be dealing with specific diseases or have identifiable characteristics (ie, use of the same program) may require different privacy safeguards [7]. Furthermore, if the association of the participant and study was revealed through this communication, the personal and integrated nature of our previous contact with the participant and our study’s purpose were considered in weighing research utility with participant confidentiality. Messages to participants included sufficient information to potentially confirm their participation (asking for confirmation of the details we found through Facebook) but not lead to involuntary disclosure of additional personal information through social media. In addition, conversations were moved off of social media once a participant had been engaged.

The All Our Families study was approved by the Child Health Research Office, Alberta Health Services, and the Conjoint Health Research Ethics Board of the University of Calgary. Written informed consent for the initial study and follow-up was obtained from the study participants at the time of recruitment, who were also provided copies for their records. Although this consent did include permission to contact for future studies, it did not specify the use of social media as a mode of contact. Additional ethical approval for this participant re-engagement was obtained, considering the specific privacy and confidentiality concerns regarding the use of social media. Data used for identifying participants were stored and analyzed on the 256-bit encrypted server at the University of Calgary, and only researchers named in the ethics file and those who sign confidentiality agreements have access to these data.

### Data Analysis

For the purpose of this analysis, participants were considered to be identified on Facebook if their identity could be confirmed on their profile. Participants were considered contacted if their privacy settings allowed for a friend request and a personal message to be sent. If a participant accepted the study’s friend request or replied to any of the messages, they were considered as being reconnected with the study. Finally, if a participant responded to the messages and provided their up-to-date contact information, they were considered as being re-engaged with the study.

Bivariate analyses were used to compare demographic variables of active study participants and participants considered lost to follow-up, using chi-square or Fisher exact tests. Bivariate analyses were also used to compare those whose Facebook profiles were identified compared with those whose could not be identified. A *P* value of less than .05 was considered statistically significant. Statistical analyses were performed using SPSS version 23 (SPSS Inc).

## Results

### Engagement

Data collection was initiated in June 2016 and ended in August 2016. Of the 237 All Our Families participants considered lost to follow-up, 47.7% (113/237) of participant profiles were identified using Facebook. Among the 113 participant profiles that were identified, 77.0% (87/113) of participants (those who accepted the friend request) were sent messages through the Facebook messenger app. Moreover, 32.7% (37/113) of participants responded to the sent messages, and 17.7% (20/113) of these participants were re-engaged in the study’s follow-up.

### Participant Characteristics

To understand the sociodemographic differences between active participants (n=2590) and participants considered lost to follow-up (n=237), baseline data were compared on the following variables: maternal age, marital status, born in Canada, household income, education status, ethnicity, and language spoken at home (Table 1). Participants considered lost to follow-up when compared with continuing participants were younger (*P*=.003), single (*P*=.023), had less household income (*P*<.001), lower education (*P*<.001), and were more likely to identify as ethnic (*P*=.015).

No significant differences were found between those whose Facebook profiles were identified (n=113) and those whose could not be identified (n=124; Table 2).

**Table 1:** Characteristics of continuing and lost to follow-up participants.

Demographic characteristics (at recruitment)		Was this participant identified as lost to follow-up?		*P* value
		No (n=2590^a^), n (%)	Yes (n=237^a^), n (%)	
**Maternal age (years)**				.003^b^
	Less than 35	2012 (77.68)	201 (88.2)	
	35 or older	503 (19.42)	27 (11.8)	
**Marital status**				.02^b^
	Married or common law	2449 (95.22)	213 (91.8)	
	Other	123 (4.78)	19 (8.2)	
**Born in Canada**				.10
	Yes	2052 (79.72)	175 (75.1)	
	No	522 (20.28)	58 (24.9)	
**Household income**				<.001^b^
	<Can $60,000	376 (15.09)	62 (27.4)	
	Can $60,000 or greater	2115 (84.91)	164 (72.6)	
**Education**				<.001^b^
	High school or less	228 (8.88)	59 (25.2)	
	Some or completed postsecondary	2341 (91.12)	175 (74.8)	
**Self-identified ethnicity**				.02^b^
	Minority	501 (19.49)	61 (26.2)	
	White	2069 (80.51)	172 (73.8)	
**Language spoken at home**				.42
	English	2298 (89.28)	204 (87.6)	
	Other	276 (10.72)	29 (12.4)	

^a^Small variations in totals may exist because of missing data (less than 3%).

^b^Indicates statistical significance (*P*<.05).

**Table 2:** Characteristics of participants identified on Facebook.

Demographic characteristics (at recruitment)		Was this participant identified on Facebook?		*P* value
		No (n=124^a^), n (%)	Yes (n=113^a^), n (%)	
**Maternal age (years)**				>.99
	Less than 35	104 (88.1)	97 (88.2)	
	35 or older	14 (11.9)	13(11.8)	
**Marital status**				.69
	Married or common law	111 (92.5)	102 (91.1)	
	Other	9 (7.5)	10 (8.9)	
**Born in Canada**				.57
	Yes	89 (73.6)	86 (76.8)	
	No	32 (26.4)	26 (23.2)	
**Household income**				.74
	<Can $60,000	31 (26.5)	31 (29.4)	
	Can $60,000 or greater	86 (73.5)	78 (71.6)	
**Education**				.5
	High school or less	33 (27.0)	26 (23.2)	
	Some or completed postsecondary	89 (73.0)	86 (76.8)	
**Self-identified ethnicity**				.49
	Minority	34 (28.1)	27 (24.1)	
	White	87 (71.9)	85 (75.9)	
**Language**				.24
	English	103 (85.1)	101 (90.2)	
	Other	18 (14.9)	11 (9.8)	

^a^Small variations in totals may exist because of missing data (less than 3%).

^b^Not applicable.

## Discussion

### Principal Findings

This study has 2 key findings. First, Facebook is a feasible way to identify participants who are lost to follow-up even without prior collection of Facebook identifiers. Using the social networking site, 48% (n=113) of lost to follow-up participants were identified and 17% (n=20) of those identified were re-engaged in the study’s follow-up. Finding and contacting participants who are lost to follow-up often depends upon collection of detailed information at the time of recruitment [18]. By using only the participant information collected at recruitment (ie, first and last name, email, and alternative contacts), almost half of the participants who were lost to follow-up were identified. The authors recognized that the privacy norms surrounding communication through social media differ from traditional means of communication by email or telephone. Although this study did not encounter negative perceptions of our use of social media from participants, the concept of privacy within social media norms is situationally dependent, and researchers must understand the context surrounding perceived privacy violations when using social media within their research [24].

Second, Facebook can assist with identifying and re-engaging participants across sociodemographic strata and may be particularly valuable for certain populations, such as those who are younger. In pregnancy cohorts, participants who are lost to follow-up are often those who are younger, unmarried, have a lower household income, less education, and more likely to self-identify as a self-identified ethnic minority compared with active participants [2]. These sociodemographic characteristics are consistent with other studies, where more vulnerable groups are more susceptible to be lost to follow-up [25,26]. Although the definition of vulnerability varies depending on locality, comparative to the relatively affluent cohort population, vulnerability in this study was defined as being younger (aged less than 35 years), lower income (<Can $60,000), or self-identified ethnic minority or not born in Canada. Of note, among those lost to follow-up, there were no sociodemographic differences between those who were identified through Facebook and those who were not, implying the feasibility of using Facebook to connect with participants without bias.

Incorporation of Facebook in the study protocols was time-efficient, and therefore cost-efficient, in comparison with other re-engagement protocols. Moreover, 1 team member actively searched, identified, and contacted each of the 237 participants in 1 week (35 hours or 2100 min), with minimal weekly follow-up messages. Approximately 10 min were spent per participant, and almost half of the participants were identified. Comparatively, the cohort staff spend 20 min per participant on phone calls to participants and alternate contacts, sending emails, and sending request letters via mail. In the case of lost to follow-up participants, *traditional* methods were ineffective as the required information (phone numbers, mailing address, and email) was out-of-date.

### Strengths and Limitations

Although this study was able to use Facebook to identify participants without a priori data collection, collection of Facebook-specific identifiers at study outset is recommended, if possible. Facebook’s privacy settings are continually changing to keep up with the public demand for privacy and security of data, and it may become more difficult to use Facebook to reconnect with participants. Unlike phone or email, Facebook URLs are associated with longer periods of use [12]. Collection of Facebook ID at the time of recruitment would expedite the process of connecting with participants and would increase confidence that the profile corresponds to the study participant. Researchers using any type of social media to engage participants should remain vigilant to the dynamic nature of privacy settings on social media platforms and would be encouraged to utilize social media early on in the study timeline as part of their knowledge dissemination strategy. This method should be adapted depending on the demographics and subject matter of the particular study. The All Our Families study is a population-based cohort, where eligibility does not imply specific personal health information, and future studies should alter the privacy measures to address the sensitivity of information being exchanged. For this study, Facebook was the best social media platform to use as the median age of the All Our Families cohort and the typical age of Facebook users overlapped [23]. However, when looking at a younger cohort, other social networking sites should be considered as they may be of greater benefit. When considering which social media site to use, the risks and benefits of each were weighed. The advantage of an open platform such as Twitter is that anyone can follow and search for users, whereas a closed platform such as Facebook allows users more control over who can view their profile. Both platforms allow direct messaging, and the accessibility of this messaging is determined by the profile owner [27]. The user interface of Twitter is more accessible to the public, and its intended use is for online social networking and microblogging. Conversely, Facebook’s functionality is more diverse and provides stronger privacy and security measures at the control of the user [28]. The decision to use a closed platform such as Facebook, rather than an open platform such as Twitter, was to ensure the privacy and confidentiality of the participants was protected, even if the risk of potential disclosure of personal information was low [29].

Facebook’s real name policy was used in this study to identify participants. Although some marginalized communities may prefer to use pseudonyms or other ways of remaining anonymous, this likely represents a small proportion of the study’s sample based off a previous cohort analysis [16]. Under this assumption, our method would lead to minimal bias in recontacting the participants from this particular study. Moreover, those identified through Facebook were compared with those not identified through Facebook based on demographic characteristics to assess selection bias. However, in utilizing the real name policy, our results may not be as generalizable to studies who work with vulnerable or marginalized populations, and the implications of this policy should be considered dependent on the specific demographics of the study.

The use of social media to re-engage participants brings in new ethical considerations, and early incorporation of possible social media use in the informed consent process is recommended. Social media technology is innovative for recruitment and retention as it encourages the free sharing of information through an interactive and expanding platform. Although all of the participants agreed to participate in the follow-up studies and signed consent forms allowing us to contact them in the future, explicit informed consent from participants at study outset for use of social networking sites for both knowledge dissemination as well as participant communication would reduce the likelihood of participants finding this form of contact intrusive and would provide support for ethical approval. This study had some limitations, including a small sample size. Only 237 participants were identified as lost to follow-up after the 7th wave of data collection in the All Our Families pregnancy cohort. This sample size limits the statistical power when determining differences between those found and those not found. However, when considering the literature surrounding finding, contacting, and re-engaging lost to follow-up participants, this study’s sample size is consistent with other studies [11,26].

An additional limitation is that only 1 social media platform was used to recontact participants, and these efforts were limited to a 3-month period. This proposed method of contacting lost to follow-up participants using Facebook was initiated in June 2016 and completed in August 2016, for a total data collection period of 3 months. The short time frame of this study’s social media presence may have limited the effectiveness in contacting and re-engaging lost to follow-up participants, especially considering the summer season. Study participants are mothers of school-aged children, and this same protocol at another time of year may increase the likelihood of contacting and re-engaging participants. In addition, the study’s Facebook profile was created specifically for this study in May 2016. Had Facebook or other social networking sites been integrated earlier into the study protocol, it may have been more successful at re-engaging lost-to-follow-up participants or reducing attrition over the 7 waves of follow-up.

The dynamic nature of privacy settings within social media sites was a further limitation to this study. At the time of the study, a friend request was necessary preceding a personalized message, as messages before friend requests were relegated to an archived message folder. Messages in the archive folder do not prompt a notification and were less accessible; however, once a friend request was accepted, a personalized message would be sent to the messenger’s standard inbox.

The results from this study demonstrated that social networking sites, such as Facebook, should be included in the development of retention practices and can be implemented at any point in cohort follow-up.
